# An overview of the development of combined oral contraceptives containing estradiol: focus on estradiol valerate/dienogest

**DOI:** 10.3109/09513590.2012.662547

**Published:** 2012-04-02

**Authors:** Franca Fruzzetti, Florence Trémollieres, Johannes Bitzer

**Affiliations:** 1 Department of Obstetrics and Gynecology, Ospedale S. Chiara, Pisa, Italy; 2 Centre de Ménopause, Hôpital Paule de Viguier, Toulouse, France; 3 Universitätsspital Basel, Frauenklinik, Basel, Switzerland

**Keywords:** Contraceptives, oral, estradiol, estrogens, menstruation

## Abstract

Natural estrogens such as estradiol (E_2_) or its valerate ester (E_2_V) offer an alternative to ethinyl estradiol (EE). E_2_-containing combined oral contraceptives (COCs) have demonstrated sufficient ovulation inhibition and acceptable contraceptive efficacy. However, earlier formulations were generally associated with unacceptable bleeding profiles. Two E_2_V-containing preparations have been approved to date for contraceptive use: E_2_V/cypro-terone acetate (CPA) (Femilar®; only approved in Finland and only in women >40 years or women aged 35–40 years in whom a COC containing EE is not appropriate) and E_2_V/dienogest (DNG; Qlaira®/Natazia®). The objective of the current review is to provide an overview of the development of COCs containing natural estrogen, highlighting past issues and challenges faced by earlier formulations, as well as the current status and future directions. The majority of information to date pertains to the development of E_2_V/DNG.

## Introduction

Combined oral contraceptives (COCs) consist of estrogen and progestogen components. Although contraceptive effects can largely be achieved with progestogen alone, as is the case for progestogen-only pills, the estrogen component in COCs avoids symptoms of hypoestrogenism [1], enhances contraceptive efficacy and helps regulate bleeding. Although a number of proges-togens have been introduced into clinical practice over the last five decades since the introduction of COCs [2–5], the estrogen component has remained predominantly ethinyl estradiol (EE). This reliance on EE to date is due mainly to its good oral bioavailability (38–48%) [6] relative to other estrogens. Recently, a new COC containing estradiol valerate (E_2_ V) combined with dienogest (DNG) has been approved for contraceptive use worldwide.

The objective of the current review is to provide an overview of the development of COCs containing natural estrogen, highlighting past issues and challenges faced by earlier formulations, as well as the present status and future directions.

## Past

### Reducing the estrogen dose

In 1970, Inman and colleagues demonstrated that COC use was associated with an increased risk of thromboembolic disease [7]. Thrombotic risk was attributed largely to the estrogen component, and increased with increasing doses of estrogen (at that time mestranol or EE) [7].

Efforts were therefore made to reduce the EE dose in COCs. These dose reductions have been highly successful in reducing the risk of venous thromboembolism (VTE); current estimates put the incidence of VTE at between 8 and 10 per 10,000 women-years in users of COCs containing <50 μg of EE, compared with 4.7 per 10,000 women-years in non-pregnant, non-COC users and around 20 per 10,000 women-years during pregnancy and the post-partum period [8].

Recent years have seen further reductions in the EE dose, to 20 μg and even 15 μg. However, these low doses have been associated with higher rates of discontinuation from clinical trials (mainly due to adverse events including bleeding) and bleeding disturbances (amenorrhea/infrequent bleeding, irregular, prolonged or frequent bleeding or spotting) compared with higher doses of EE [9]. In particular, preparations containing EE 15 μg have a somewhat higher incidence of breakthrough bleeding and/or spotting than COCs containing EE 20 μg [10], and may be associated with premature discontinuation because of bleeding irregularities [11]. These observations, together with the finding that even EE doses as low as 10 μg have been associated with negative effects on hemostatic surrogate parameters [12], mean that it is unlikely that a COC containing lower EE dosages will be well accepted.

### Estradiol versus EE: pharmacological effects

**Figure 1:**
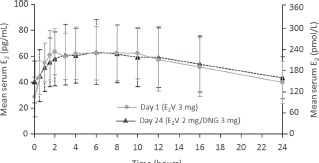
Mean serum estradiol (E_2_) concentration over 24 h following oral administration of estradiol valerate (E_2_V)/dienogest (DNG) [17]. Zeun S, et al., Eur J Contracept Reprod Health Care, 2009;14(3):221–32, copyright© 2009, Informa Healthcare. Reproduced with permission of Informa Healthcare.

**Figure 2:**
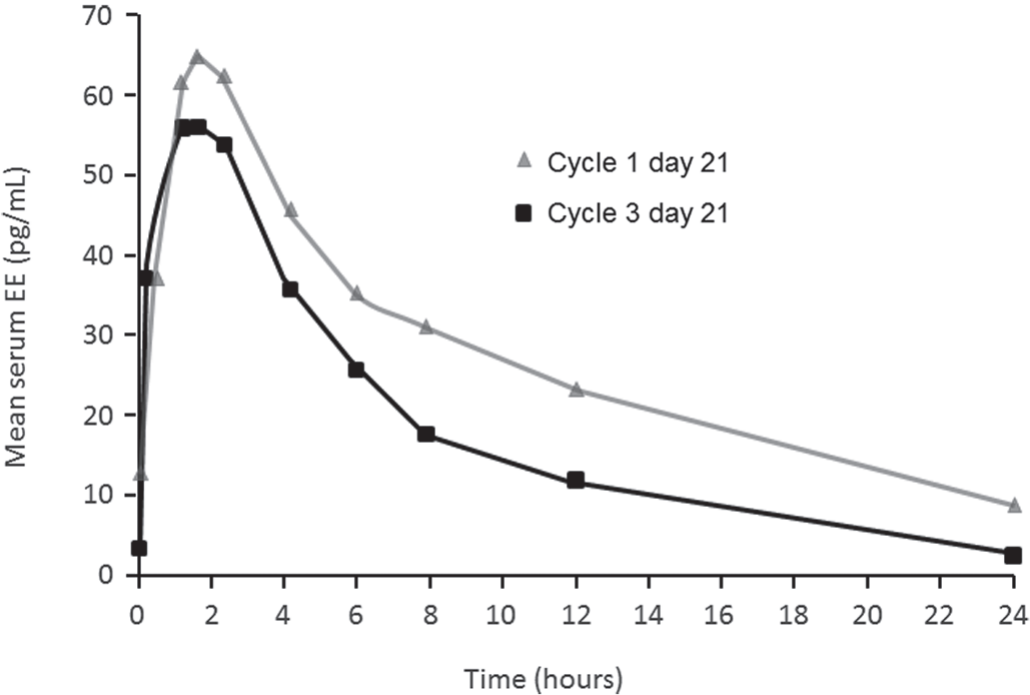
Mean ethinyl estradiol (EE) concentration over 24 hours following oral administration of EE 20 μg/levonorgestrel (LNG) 100 μg [18]. Endrikat J, et al., Eur J Contracept Reprod Health Care, 2002;7(2):79–90, copyright© 2002, Informa Healthcare. Reproduced with permission of Informa Healthcare.

Exogenously administered estradiol is chemically identical to endogenous 17β-estradiol (E_2_), the most potent of the natural estrogens [13]. In the past, a major obstacle to using E_2_ in hormonal contraceptives was its relative inactivity when administered orally [13]. As outlined previously, EE was first used in COCs because of its good oral bio availability (38–48%) [6] compared with E_2_ (5%) [14]. Different approaches have been undertaken to overcome the low bio availability of E_2_, including micronization and esterification [15]. E_2_V is the valerate ester of natural E_2_. The estrogenic effects and pharmacokinetic profile of E_2_V and E_2_ are comparable as E_2_V is rapidly converted to E_2_ in the gut and liver [16]. Following oral administration of E_2_V (in combination with DNG), serum concentrations of E_2_ remain fairly stable during a 24-h period (Figure 1) [17]. In contrast, peak serum EE levels following administration of EE (in combination with levonorgestrel [LNG]) are reached after 1.5 h and reduce thereafter (Figure 2) [18].

At equivalent dosages (i.e. E_2_ 2 mg has biologic effects that are equivalent to EE 4–20 μg, depending on the target organ) E_2_ has been shown to have a lesser impact on metabolic and hepatic parameters than EE in several studies. This is manifested in a more favorable effect of E_2_ versus EE on lipids [19] and a reduced effect of E_2_ versus EE on the synthesis of hepatic proteins, including sex hormone-binding globulin (SHBG) and angiotensinogen [6,20,21]. In addition, E_2_ appears to have a reduced impact on markers of hemostasis than EE [12,22,23].

### Clinical experience with estradiol-containing COCs

The design and results of clinical studies investigating E_2_-containing COCs are shown in Table I [24–43]. These studies have shown that, in terms of efficacy, these preparations demonstrate sufficient ovulation inhibition and an acceptable level of contraceptive efficacy. Seven studies examined preparations that no longer appear to be in development [25,29,36,38–42]. Three of these studies investigated the combination of E_2_ with nore-thisterone, with or without the addition of E_3_ [25,39,40,42]. In all studies, the E_2_ dose was 4 mg, the norethisterone dose was 3 mg, and, where applicable, the E_3_ dose was 2 mg. Effective inhibition of ovulation was noted with all regimens, but unacceptable bleeding profiles limited the use of this combination [25,39,40,42]. Four of the studies examined the combination of E_2_ with desogestrel (DSG) [29,36,38,41]. The dose of E_2_ varied from 1 to 3 mg, and the dose of DSG was 150 μg in all studies. The studies were very small (20–31 subjects). As described previously, ovulation inhibition was noted in all studies, but this benefit was outweighed by an unacceptable bleeding profile [29,36,38,41]. Similar trends were observed when the combination of E_2_ cyclo-octyl acetate/DSG was examined [38].

**Table I:** Summary of clinical studies in contraception involving combined oral contraceptives (COC) that incorporated estradiol (E_2_) rather than ethinyl estradiol (EE)

Reference	Study design	*n*	Intervention	Key results
*COCs apparently no longer in development*				
Astedt et al. 1979 [25]	Randomized	215	*21/7 regimen for 12 cycles*	No pregnancies.
	Triple-blind		Micronized E_2_ 4 mg/norethisterone 3 mg (Netagen 403) for 21 days followed by 7-day pill-free period	No thrombotic events.
				No thrombotic events.
			*OR*	Discontinuation rates were similar between treatment groups (Netagen 403: *n* = 39; Netagen 423: *n* = 31; Netasyn: *n* = 35)
			Micronized E_2_ 4 mg + E3 2 mg/norethisterone 3 mg (Netagen 423) for 21 days followed by 7-day pill-free period	The main reasons for discontinuation: amenorrhea and weight gain with Netagen 403; intermenstrual spotting with Netagen 423; nausea and weight gain with Netasyn.
			*OR*	
			EE 50 μg/norethisterone 3 mg (Netasyn) for 21 days followed by 7-day pill-free period	
Serup et al. 1979 [40], Serup et al. 1981 [39]	Randomized	111	*21/7 regimen for 12 cycles*	No pregnancies.
	Double-blind		E_2_ 4 mg/E3 2 mg/norethisterone acetate 3 mg for 21 days followed by 7-day pill-free period	
			*OR*	
			EE 50 μg/norethisterone acetate 3 mg for 21 days followed by 7-day pill-free period	Spotting and breakthrough bleeding were significantly more common with natural estrogen than with EE (*p* < 0.01) and led to significantly more discontinuations (*p* < 0.001). Bleeding irregularities did not subside during the study.
World Health Organization 1980 [42]	Randomized	925	*21/7 regimen for 12 cycles*	Annual failure rate was approximately 1 per 100 women in each treatment arm.
	Double-blind		Micronized E_2_ 4 mg + E3 2 mg/norethisterone acetate 3 mg for 21 days followed by 7-day pill-free period	Menstrual irregularities, including all menstrual complaints, amenorrhea, light bleeding and spotting, were significantly more common with natural estrogens than with EE (*p* < 0.01).
			*OR*	Discontinuation rate at 1 year: 48.4% for EE and 51.5% for natural estrogens.
			EE 50 μg/norethisterone acetate 3 mg for 21 days followed by 7-day pill-free period	Discontinuation for menstrual irregularities was significantly higher with natural estrogens (48 *vs.* 13 cases; *p* < 0.001). Menstrual irregularities were more common with natural estrogens during cycles 1–3, 10 and 12.
Schubert et al. 1987 [38]	Randomized	10	*21/7 regimen for 1 cycle*	Follicular development and ovulation were inhibited.
	Blinding not specified		E_2_ cyclo-octyl acetate 0.5 mg/DSG 150 μg for 21 days followed by 7-day pill-free period	An unacceptable bleeding profile was observed – all women experienced breakthrough bleeding.
			*OR*	
			E_2_ cyclo-octyl acetate 0.5 mg/DSG 150 μg for 21 days followed by DSG 30 μg for 7 days	
Wenzl et al. 1993 [41]	Single-arm	20	*21/7 regimen for 2 cycles*	Ovulation inhibition was observed in all women.
	Open-label		Micronized E_2_ 1 mg/DSG 150 μg for 21 days followed by a 7-day pill-free period	Approximately two-thirds of women experienced withdrawal bleeding in each cycle.
				Bleeding during the treatment cycles was heavier and longer than the pre-treatment cycle.
				Two women discontinued due to unacceptable bleeding in cycle 1.
Csemiczky et al. 1996 [29]	Randomized	29	*21/7 regimen for 2 cycles*	Ovulation was suppressed in all women.
	Double-blind		Micronized E_2_ 3 mg/DSG 150 μg for 21 days followed by DSG 30 μg for 7 days DSG 30 μg for 7 days	Withdrawal bleeding occurred in 81% of women
			*OR*	Breakthrough bleeding and spotting were more common in DSG group (46.2% vs. 26.2%).
			Micronized E_2_ 3 mg/DSG 150 μg for 21 days followed by placebo for 7 days	No discontinuations due to bleeding disturbances were observed.
Kivinen and Saure 1996 [36]	Randomized	31	*21/7 regimen for 6 cycles*	Ovulation was suppressed in all women.
	Open-label		A: Micronized E_2_ 1.5 mg/DSG 150 μg for 21 days followed by 7-day pill-free period	The mean number of bleeding and/or spotting days per cycle was 11.2 for group A, 6.4 for group B and 6.2 for group C.
			*OR*	The mean number of unexpected spotting/bleeding days per cycle was 4.3, 1.9 and 2.2, respectively.
			B: Micronized E_2_ 3 mg/DSG 150 μg for 21 days followed by 7-day pill-free period	Discontinuations occurred in 3, 3 and 5 women, respectively
			*OR*	Reasons for discontinuation: unexpected bleeding and irritability; bleeding, nausea and headache; bleeding, breast tenderness, irritability.
			C: Micronized E_2_ 3 mg/DSG 150 μg for 21 days followed by E_2_ 1 mg for 7 days	
COCs in development or commercially available				
Hirvonen et al. 1988 [33]	Randomized	50	*21/7 regimen for 6 cycles*	Ovulation was inhibited in all women (except for one woman who ovulated during the first treatment cycle) in the E_2_V/CPA group. In the E_2_V/norethisterone group, ovulation occurred in 8 women. One additional woman in this group ovulated during all treatment cycles.
	Double-blind	E_2_V 1 mg/CPA 1 mg for 10 days followed by E_2_V 2 mg/CPA 2 mg for 11 days followed by 7-day pill-free period (approved only in women >40 years or women 35–40 years in whom a COC containing EE is not appropriate)	Menstrual blood loss reduced in all women in the E_2_V/CPA group. In the E_2_V/norethisterone group, menstrual blood loss reduced in 40% and increased in 10% of women.	
			*OR*	The total number of bleeding days reduced with E_2_V/CPA and increased with E_2_V/norethisterone.
			E_2_V 1 mg/norethisterone 1 mg for 10 days followed by E_2_V 2 mg/norethisterone 2 mg for 11 days followed by 7-day pill-free period (not commercially available)	
Hirvonen et al. 1995 [32]	Single-arm	288	*21/7 regimen for 12 cycles*	Ovulation was inhibited in 95% of women.
	Open-label		E_2_V 1 mg/CPA 1 mg for 10 days followed by E_2_V 2 mg/CPA 2 mg for 11 days followed by 7-day pill-free period (approved only in women >40 years or women 35–40 years in whom a COC containing EE is not appropriate)	Cumulative pregnancy rate was 0.4%.
				12.
				Bleeding became less frequent over time.
				Dysmenorrhea subsided over time.
Duijkers et al. 2010 [30]	Randomized	48	*24/4 or 21/7 regimen for 6 cycles*	Ovulation was suppressed in all women.
	Open-label		E_2_ 1.5 mg/NOMAC 2.5 mg for 24 days followed by 4-day pill-free period	Reductions in follicle size were observed with both treatments, from 19.3 mm to 6.9–8.2 mm with E_2_/NOMAC and from 19.6 mm to 7.4–10.8 mm with EE/drospirenone.
			*OR*	
			EE 30 μg/drospirenone 3 mg for 21 days followed by 7-day pill-free period	
Chabbert-Buffet et al. 2011 [27]	Randomized	41	*21/7 regimen for 1 cycle*	Ovulation was suppressed in all treatment groups.
	Double-blind		E_2_ 1.5 mg/NOMAC 0.625 mg for 21 days followed by 7-day pill-free period	The lowest plasma E_2_ levels were observed with NOMAC 2.5 mg.
			*OR*	The addition of E_2_ 1.5 mg to NOMAC 2.5 mg resulted in statistically significant increases in E_2_ levels and decreases in mean follicle-stimulating hormone and luteinizing hormone levels.
			E_2_ 5 mg/NOMAC 1.25 mg for 21 days followed by 7-day pill-free period	
			*OR*	
			E_2_ 1.5 mg/NOMAC 2.5 mg for 21 days followed by 7-day pill-free period	
			*OR*	
			NOMAC 2.5 mg for 21 days followed by 7-day pill-free period	
Christin-Maitre et al. 2011 [28]	Randomized	77	*21/7- or 24/4-day regimen for 3 cycles*	Ovulation was inhibited with both regimens.
	Double-blind		E_2_ 1.5 mg/NOMAC 2.5 mg for 24 days followed by E_2_ for 4 days	The largest follicular diameter was significantly smaller and mean follicle stimulating hormone levels were significantly lower with the 24/4 regimen than the 21/7 regimen (*p* < 0.05).
			*OR*	The duration of total and withdrawal bleeding was significantly lower with the 24/4 regimen than the 21/7 regimen (*p* < 0.05).
			E_2_ 1.5 mg/NOMAC 2.5 mg for 21 days followed by E_2_ for 7 days	
Gaussem et al. 2011 [43]	Randomized	90	*21/7- or 24/4-day regimen for 3 cycles*	This study compared the hemostatic effects of E_2_V/DNG with those of E_2_/NOMAC. E_2_V/DNG and E_2_/NOMAC were associated with similar effects on sex hormone-binding globulin, prothrombin fragment 1+2, fibrinogen and thrombin generation.
	Double-blind		E_2_ 1.5 mg/NOMAC 2.5 mg for 24 days followed by a 7-day pill-free period	
			*OR*	Bleeding was assessed as a secondary outcome in this study. The duration of total bleeding, withdrawal bleeding and breakthrough bleeding appeared to be lower with E_2_/NOMAC than with EE/LNG.
			EE 20 μg/LNG 100 μg for 21 days followed by a 7-day pill-free period	
Hoffman et al. 1998 and 1999 [34,35]	Randomized	20	*28-day regimen for 6 cycles*	Ovulation was inhibited with both regimens.
	Open-label		E_2_V 2 mg/DNG 2 mg for 24 days followed by E_2_V 2 mg for 4 days (development regimen within Qlaira® development program)	Withdrawal bleeding occurred in 91% of women in the first group and 87% of women in the second group.
	Pilot study			Irregular bleeding was observed in 60–75% of women.
			*OR*	
			E_2_V 2 mg/DNG 2 mg for 7 days followed by E_2_V 4 mg/DNG 2 mg for 14 days followed by E_2_V 2 mg for 7 days (development regimen within Qlaira® development program)	
Hoffman et al. 1998 and 1999 [34,35]	Not given	100	*25/3 regimen for 6 cycles*	No pregnancies.
	Pilot study		E_2_V 3 mg for 3 days followed by E_2_V 2 mg/DNG 1 mg for 4 days followed by E_2_V 2 mg/DNG 2 mg for 16 days followed by E_2_V 1 mg for 2 days followed by placebo for 3 days (development regimen within Qlaira® development program)	By cycle 6, regular bleeding was noted in 97% of cycles.
				Intermenstrual bleeding was observed in 30% of cycles during cycles 1–3 and 15.6% of cycles at cycle 6.
Endrikat et al. 2008 [31]	Randomized	Study 1: 192	*Various dynamic dosing regimens containing E_2_V/DNG for 3 cycles*	The following regimen contained the lowest dose of DNG necessary for suppression of ovulation (defined as ovulation rate <5% with an upper limit of the 95% CI of <10% in cycle 2): E_2_V 3 mg alone for 2 days followed by E_2_V 2 mg/DNG 2 mg for 5 days followed by E_2_V 2 mg/DNG 3 mg for 17 days followed by E_2_V 1 mg alone for 2 days followed by placebo for 2 days.
	Open-label	Study 2: 203		
				In cycle 2, the above regimen was associated with a Hoogland score of 5 or 6 in three of 96 women (3.1%; 90% CI = 0.2–6.1%).
				No safety concerns were raised with any of the regimens studied.
Ahrendt et al. 2008 [24]	Randomized	798	*26/2 or 21/7 regimen for 7 cycles*	One pregnancy occurred in the EE/LNG group.
	Double-blind		E_2_V 3 mg alone for 2 days followed by E_2_V 2 mg/DNG 2 mg for 5 days followed by E_2_V 2 mg/DNG 3 mg for 17 days followed by E_2_V 1 mg alone for 2 days followed by placebo for 2 days	Scheduled withdrawal bleeding occurred in 77.7–83.2% of E_2_V/DNG recipients and 89.5–93.8% of EE/LNG recipients.
				The intensity and duration of withdrawal bleeding was reduced with E_2_V/DNG compared with EE/LNG.
			*OR*	Intracyclic bleeding was similar between the two COCs (10.5–18.6% *vs.* 9.9–17.1% per cycle).
			EE 20 μg/LNG 100 μg for 21 days followed by placebo for 7 days	
Palacios et al. 2010 [37]	Single-arm	1377	*26/2 regimen for 20 cycles*	In this study in European women, this regimen was associated with an adjusted Pearl Index of 0.34.
	Open-label		E_2_V 3 mg alone for 2 days followed by E_2_V 2 mg/DNG 2 mg for 5 days followed by E_2_V 2 mg/DNG 3 mg for 17 days followed by E_2_V 1 mg alone for 2 days followed by placebo for 2 days	Overall, 2.5% of women prematurely discontinued treatment because of menstrual bleeding irregularities.
Natazia® Prescribing Information 2010 [26]	Single-arm	490	*26/2 regimen for up to 28 cycles*	In this study in North American women, this regimen was associated with an unadjusted Pearl Index of 1.64.
	Open-label		E_2_V 3 mg alone for 2 days followed by E_2_V 2 mg/DNG 2 mg for 5 days followed by E_2_V 2 mg/DNG 3 mg for 17 days followed by E_2_V 1 mg alone for 2 days followed by placebo for 2 days	

CI, confidence interval; CPA, cyproterone acetate; DSG, desogestrel; E_2_V, estradiol valerate; E3, estriol; LNG, levonorgestrel; NOMAC, nomegestrol acetate.

The underlying reasons for the unacceptable bleeding profiles observed in these studies may include an inappropriate estrogen/ progestogen ratio [36,38,41,42] or suboptimal E_2_ doses [40,42].

## Present

### Combinations of estradiol plus different progestogens

#### Estradiol/NOMAC

Nomegestrol acetate (NOMAC) is a 17-hydroxy-progesterone derivative [30]. A recent study compared E_2_ 1.5 mg/NOMAC 2.5 mg with EE 30 μg/drospirenone 3 mg in healthy women (n = 32) (Table I) [30]. In this randomized, six-cycle study, ovulation inhibition was noted in all women in both treatment groups. Ovarian suppression was similar between treatments; progesterone was fully suppressed to levels <2 nmol/L in both groups (Table I) [30]. A more recent publication noted that NOMAC 2.5 mg inhibited both ovulation and follicular maturation, and the antigonadotropic effects of NOMAC 2.5 mg were reinforced when it was combined with E_2_ 1.5 mg [27]. This dose-finding study underlines the role of the estrogen component in inhibiting ovulation. A third study assessed ovarian activity with two different E_2_ (1.5 mg)/NOMAC (2.5 mg) regimens (21/7 [*n* = 37] and 24/4 [*n* = 40]) [28]. The 24/4 regimen was associated with greater inhibition of follicular activity. A shorter duration of total and withdrawal bleeding with the 24/4 regimen compared with the 21/7 regimen was described as secondary outcome (*p* < 0.05) [28]. By cycle 3, the incidence of breakthrough bleeding was similar between regimens, but the duration of breakthrough bleeding was slightly longer with the 21/7 regimen than with the 24/4 regimen [28]. The bleeding profile was also assessed as a secondary outcome in a study comparing the hemostatic effects of E/NOMAC in a 24/4 regimen with those of EE/LNG in a 21/7 regimen. In this short-term study, also based on a limited number of participants and thus not powered to investigate the bleeding profile (*n* = 45 in each group), the duration of total bleeding, withdrawal bleeding and breakthrough bleeding appeared to be shorter with E_2_/NOMAC than with EE/LNG (Table I) [43]. Meaningful investigations of the bleeding profile of E_2_/NOMAC in a 24/4 regimen and comparisons with other combined oral formulations in a larger more diverse group of women are currently lacking. Contraceptive efficacy was not assessed in these studies.

#### Estradiol/drospirenone

Two randomized, double-blind, parallel-group Phase II studies have been completed with a COC containing E_2_/drospirenone administered in either a monophasic or triphasic regimen (dosages not defined). Both studies were conducted in healthy women aged 18–35 years. The first study (*n* = 116) assessed ovulation inhibition (ClinicalTrials.gov identifier: NCT00631124), while the second (*n* = 575) evaluated cycle control and safety (ClinicalTrials.gov identifier: NCT00653614). Results are anticipated.

#### Estradiol valerate/CPA

The combination of E_2_V and CPA has been marketed as a COC (Femilar®) in Finland since 1993, however it is only indicated in women >40 years or women aged 35–40 years in whom a COC containing EE is not appropriate. This formulation comprises E_2_V 1 mg/CPA 1 mg on days 1–10, E_2_V 2 mg/CPA 2 mg on days 11–21 and a pill-free interval on days 22–28. During 12 cycles of treatment with the E_2_V/CPA combination (*n* = 288), ovulation inhibition was observed in 95% of women. The cumulative pregnancy rate was 0.4% (Table I) [32]. Intermenstrual bleeding/spotting was observed in 35.5% of women in cycle 3 and 24.5% of women in cycle 12 [32]. Bleeding became less frequent over time in the majority of women, and dysmenorrhea subsided [32]. Similar findings were observed in a second study (*n* = 50) comparing E_2_W CPA with E_2_V/norethisterone in a biphasic regimen (E_2_V/CPA: as described above; E_2_V/norethisterone: E_2_V 1 mg/norethisterone 1 mg on days 1–10, E_2_V 2 mg/norethisterone 2 mg on days 11–21 and a pill-free interval on days 22–28) (Table I) [33]. Ovulation was inhibited in all women (except for one woman who ovulated during the first treatment cycle) in the E_2_ V/CPA group. In the E_2_V/norethisterone group, ovulation occurred in 8 women. One additional woman in this group ovulated during all treatment cycles; treatment was discontinued in this subject. Contraceptive efficacy was not assessed in this study. Menstrual blood loss was reduced in all women in the E_2_V/CPA group. However, in the E_2_V/norethis-terone group, menstrual blood loss reduced in 40% and increased in 10% of women. The total number of bleeding days reduced with E_2_V/CPA and increased with E_2_V/norethisterone (Table I) [33].

#### Estradiol valerate/DNG

The combination of E_2_V/DNG was approved as a COC in the European Union (EU) in 2008, where it is marketed as Qlaira®/Klaira®. FDA approval for Natazia® was obtained in May 2010. Qlaira® received regulatory approval in the EU for the treatment of heavy menstrual bleeding (HMB) in October 2010 and in the USA (Natazia®) in March 2012. The E_2_V/DNG combination provides early estrogenic dominance to ensure initial endome-trial proliferation and endometrial stroma stability during the progestogen-dominated mid-to-late part of the cycle [24]. DNG has potent endometrial activity [44–46] and a bioavailability of >90% after oral intake [47].

Early investigations with E_2_V/DNG employed biphasic or triphasic regimens, which provided effective ovulation inhibition but unacceptable bleeding profiles (Table I). The unacceptable bleeding profile with E_2_V/DNG in a biphasic or triphasic regimen prompted the introduction of an E_2_V/DNG combination in an estrogen step-down/progestogen step-up approach. In a pilot study in healthy women (*n* = 100), a dynamic dosing regimen (E_2_V 3 mg for 3 days, E_2_V 2 mg/DNG 1 mg for 4 days, E_2_V 2 mg/ DNG 2 mg for 16 days, E_2_V 1 mg for 2 days and finally placebo for 3 days) was associated with a far more favorable profile than the biphasic or triphasic regimens (Table I) [34,35].

**Figure 3:**
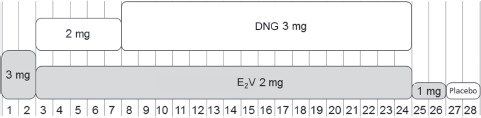
Dosing regimen of an estradiol valerate (E_2_V)/dienogest (DNG)-containing oral contraceptive administered using an estrogen step-down and progestogen step-up approach over a 28-day treatment cycle (with 26 days of active tablets). Numbers along the bottom of the figure correspond to days of the 28-day cycle.

Four variations of E_2_V/DNG in dynamic phasic regimens were investigated in two sequential Phase II studies designed to determine the optimal daily application and the required dose of DNG for effective inhibition of ovulation (Table I) [31]. In the first study it was shown that a dosing regimen that incorporated 26 rather than 25 days of active treatment was associated with greater ovulation inhibition. In the second study, which examined two 26-day regimens with doses of DNG that were distinctly increased versus those used in the first study, it was shown that a dose of DNG of 2 mg on days 3–7 and 3 mg on days 8–24 (both in combination with E_2_V 2 mg) was the lowest effective dose of DNG for efficient ovulation inhibition. This regimen comprised E_2_V 3 mg alone for 2 days, E_2_V 2 mg/DNG 2 mg for 5 days, E_2_V 2 mg/DNG 3 mg for 17 days, E_2_V 1 mg alone for 2 days then placebo for 2 days (Figure 3 and Table I). There are few data on compliance with COCs, but one would expect that reducing the hormone-free interval to only 2 days (i.e. 26 days of active treatment, 2 days of placebo [26/2 regimen]) would improve tolerability and, in turn, improve compliance.

The efficacy, bleeding profile and safety of E_2_ V/DNG in a dynamic dosing regimen has been examined in three Phase III trials (Table I). The first of these trials enrolled 1377 women aged 18–50 years and was conducted in Europe over twenty 28-day cycles [37]. All women received the regimen outlined above. The E_2_V/DNG combination was associated with an adjusted Pearl Index of 0.34 (upper limit of 95% CI = 0.73), together with good tolerability and a high degree of user satisfaction. Only 2.5% of 1377 women treated for up to 20 cycles prematurely discontinued treatment because of menstrual bleeding irregularities [37]. The second study, conducted in the USA and Canada, was an open-label, non-comparative study designed to assess the contraceptive efficacy, cycle control, safety and tolerability of E_2_ V/DNG. A total of 490 women aged 18–35 years received E_2_V/DNG for up to 28 cycles [48]. The third study, conducted in Europe, compared the E_2_V/DNG regimen with a monophasic COC (EE 20 μg/LNG 100 μg) over seven cycles (*n* = 798) [24]. Scheduled withdrawal bleeding occurred in 77.7–83.2% of E_2_V/DNG recipients and 89.5–93.8% of EE/LNG recipients. The maximum intensity of withdrawal bleeding was significantly different in women treated with E_2_V/DNG and EE/LNG; a greater proportion of women who received E_2_V/DNG versus EE/LNG experienced spotting or light bleeding and a smaller portion of women who received E_2_V/DNG versus EE/LNG experienced normal or heavy bleeding. In addition, the mean duration of withdrawal bleeding was reduced with E_2_V/DNG compared with EE/LNG (4.1–4.7 vs. 5.0–5.2 days;p < 0.05 per cycle). Intracyclic bleeding was similar between the two COCs (10.5–18.6% vs. 9.9–17.1% per cycle; *p* > 0.05) (Table I) [24].

Based on data from the three Phase III trials performed in Europe, the USA and Canada, E_2_V/DNG was associated with a typical-use Pearl Index of 0.79 (upper limit of 95% CI = 1.23) and a perfect-use Pearl Index of 0.42 (upper limit 95% CI = 0.77) in women aged 18–50 years [49]. In women aged 18–35 years, the corresponding Pearl Indices were 1.01 (upper limit 95% CI = 1.59) and 0.51 (upper limit 95% CI = 0.97) [49].

#### Pharmacokinetics of estradiol valerate/DNG

**Figure 4:**
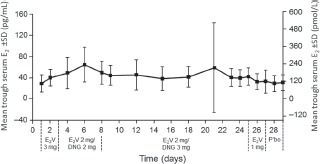
Minimum mean (standard deviation [SD]) serum concentrations of estradiol during daily administration of a 28-day oral contraceptive containing estradiol valerate (E_2_V)/dienogest (DNG) [50].

The pharmacokinetics of E_2_V/DNG were analyzed in 15 healthy women aged 18–50 years who participated in a Phase I, open-label, single-cycle study [17]. E_2_V/DNG was associated with stable serum levels of E_2_ throughout the 28-day period of treatment (Figure 4) [17,50]. Minimum mean serum E_2_ levels during E_2_V administration (days 1–26) were 33.6–64.7 pg/mL, similar to those seen in the mid-follicular phase of normal ovulatory cycles, while minimum mean serum DNG levels were 6.8–15.1 ng/mL during DNG administration (days 3–24). Minimum concentrations of DNG showed only minor accumulation within each phase of the regimen during which DNG was administered. On day 24, the geometric mean maximum concentration of DNG was 82.9 ng/mL, while the average concentration and terminal half-life were 33.7 ng/mL and 12.2 h. The median time to maximum observed drug concentration for DNG was 1.5 h. Serum SHBG concentrations increased by 40%, but remained within the normal range. Cortisol-binding-globulin levels remained essentially unchanged.

#### Metabolic and vascular effects ofestradiol valerate/DNG

Data have shown that E_2_V/DNG has an impact on various metabolic and hemostatic parameters that is comparable to or less than that of EE/LNG-containing COCs [51,52]. The effect on prothrombin and D-dimer levels is marginal [52], and the effects on high- and low-density lipoprotein cholesterol [51], insulin [51], and carbohydrate metabolism [51] are, in general, more favorable with E_2_V/DNG than with EE/LNG.

The hemostatic effects of E_2_V/DNG and E_2_/NOMAC have not been compared in a head-to-head trial; however, a comparison of data from different trials suggests that the overall magnitude of changes in hemostatic parameters during treatment with E_2_V/DNG is comparable to that reported with the E_2_/NOMAC combination [43,51,52]. For example, E_2_V/DNG and E_2_/NOMAC were associated with similar effects on SHBG, prothrombin fragment 1+2, fibrinogen and thrombin generation (either determined by the activated protein C (APC) sensitivity ratio or by APC resistance) as measured by the endogenous thrombin generation method [43,51–53]. This suggests that the hemostatic effects of COCs comprising either E_2_ or E_2_V in equimolar dosages are comparable, as one would expect.

Although these results are reassuring, one should bear in mind that the lipid, metabolic and hemostatic parameters are only surrogate markers, and have yet to be fully validated as good predictors of the occurrence of clinical events such as VTE. Furthermore, even oral intake of E_2_ is associated with a hepatic first pass effect [6,16] that may impair the biosynthesis and clearance of proteins involved in hemostasis or blood pressure regulation. Further clinical and long-term epidemiological studies of E_2_V/DNG in large populations are needed before any safety conclusions can be made. A large international prospective, controlled, non-in-terventional cohort active surveillance study (INAS-SCORE) to investigate the occurrence of cardiovascular events over a 3- to 5-year period in COC users (including E_2_V/DNG) is currently underway (ClinicalTrials.gov identifier: NCT01009684).

#### Additional non-contraceptive benefits ofestradiol valerate/DNG

E_2_V/DNG has been shown to be effective in women with HMB (defined as menstrual blood loss >80mL). The registration procedure has been successfully concluded for an indication of HMB without organic pathology in the EU, Switzerland and USA and for heavy and/or prolonged menstrual bleeding in Australia and several Latin American and Asian countries. License applications for the HMB indication have also been submitted to health authorities in other countries. Overall, E_2_V/DNG was associated with an 88% reduction in median menstrual blood loss (from 142 mL to 17 mL/cycle) after 6 months of treatment, compared with a 24% reduction with placebo (from 154 mL to 117 mL/cycle) [54]. Reductions in MBL volume were rapid and sustained and were deemed clinically meaningful [54].

The efficacy of this combination in HMB is thought to be due to its unique dosing regimen, which enables estrogen dominance during the early part of the cycle and progestogen dominance in the late part of the cycle [24]. Women receive 26 days of E_2_V, which supports endometrial stability [17], and 22 days of DNG, a progestogen with high endometrial potency [44–46].

In summary, the development of dynamic regimens has yielded acceptable bleeding patterns whilst maintaining a reliable level of contraceptive efficacy. Data have shown this regimen to provide effective ovulation inhibition with an acceptable level of cycle control in healthy women.

## Future directions

In modern contraception, one focus lies in additional health benefits. There is, therefore, a great deal of interest in whether E_2_-containing COCs have known or even new benefits. In order to establish these benefits, extensive efforts have been put into the development program for E_2_V/DNG, with one of the most comprehensive Phase Illb clinical programs for a COC.

The HARMONY I and II studies are multicenter, randomized, double-blind, active control group studies comparing the efficacy of the E_2_V/DNG combination with that of EE/norg-estimate (ClinicalTrials.gov identifier: NCT00754065) or EE/LNG (ClinicalTrials.gov identifier: NCT00778609) for the treatment of hormone withdrawal-associated symptoms (HWAS), including headache, pelvic pain and bloating. The studies aim to demonstrate the superiority of E_2_V/DNG over the comparators with regards to HWAS improvement after six cycles of treatment. Both studies are underway and results are anticipated in 2011. It is expected that E_2_V/DNG will have a beneficial effect on cycle-related hormone withdrawal symptoms owing to less hormonal fluctuations. Specifically, E_2_V/DNG is associated with levels of E_2_ that are stable and are comparable to those during the first week of the follicular phase of a spontaneous menstrual cycle [17]. In addition, the hormone-free interval with E_2_V/DNG (of 2 days) is shorter than that of conventional COCs (7 days). It has been shown previously that hormone-related symptoms are worse during the 7-day hormone-free interval than during active treatment [55], and shortening the hormone-free interval from 7 to 4 days may decrease the number of days of symptoms typically associated with hormone withdrawal [55–57].

The STABLE study (ClinicalTrials.gov identifier: NCT 00764881) aims to establish the non-inferiority of E_2_V/DNG over EE/LNG for improving libido in women with OC-associated female sexual dysfunction (FSD). Women presenting with OC-associated FSD are often switched to a COC containing EE/LNG, in the belief that the partial androgenic effects of LNG will alleviate FSD symptoms. STABLE is, to our knowledge, one of the first, if not the first, comprehensive, randomized controlled trial with a comparator arm, using validated questionnaires to investigate the effects of different COCs on OC-associated FSD. The benefit of E_2_V/DNG on libido in women with OC-induced FSD is thought to be due to the low impact of E_2_V/DNG on SHBG and its beneficial effect on vaginal cell maturation, demonstrating the complex and multifac-torial nature of OC-associated FSD. Therefore, effects other than just the androgenic nature of the progestogen should also be taken into account in women with OC-associated FSD.

The CALM study (ClinicalTrials.gov identifier: NCT00909857) is investigating the effect of E_2_V/DNG on primary dysmenorrhea. In this multicenter, randomized, double-dummy, parallel-group study, women will receive E_2_V/DNG or EE/LNG for three cycles. Results for both STABLE and CALM are expected in 2011.

## Conclusions

The quest to identify an effective, well tolerated COC using natural estrogens, such as E_2_, has encompassed a number of clinical trials over several decades. Earlier attempts to use E_2_ in COCs explored various doses, progestogens and regimens, but yielded unfavorable results in terms of bleeding and cycle control. Recent data suggest that the combination of E_2_/NOMAC may yield promising results. Further studies are, however, needed.

Two E_2_V-containing COCs are currently available; E_2_V/CPA has been marketed as Femilar® in Finland since 1993 (only indicated in women >40 years or women aged 35–40 years for whom a COC containing EE is not appropriate), and E_2_V/DNG has recently been launched in the EU as Qlaira®/Klaira® and in the USA as Natazia®. There are extensive data available regarding the E_2_V/DNG combination, which is administered in a dynamic dosing regimen. This combination has been shown to provide women with reliable contraceptive efficacy whilst maintaining acceptable cycle control. It is also effective for the treatment of HMB, and may offer additional non-contraceptive benefits, such as improvements in OC-associated FSD, dysmenorrhea and hormone withdrawal-associated adverse events.
